# Sacral osteomyelitis as a rare cause of anorectal pain several years following treatment for rectal carcinoma

**DOI:** 10.1093/omcr/omac037

**Published:** 2022-04-19

**Authors:** Darren C R Fernandes, Sangeetha Srinivasan, Hubert Jervoise N Andreyev

**Affiliations:** Department of Gastroenterology, Lincoln County Hospital, United Lincolnshire NHS Trust, Lincoln, UK; School of Health and Social Care, Community and Health Research Unit, University of Lincoln, Lincoln, UK; Department of Radiology, Lincoln County Hospital, United Lincolnshire NHS Trust, Lincoln, UK; Department of Gastroenterology, Lincoln County Hospital, United Lincolnshire NHS Trust, Lincoln, UK; Biomedical Research Centre, Nottingham Digestive Diseases Centre, School of Medicine, University of Nottingham, Nottingham, UK

## Abstract

A 66-year-old man was treated for a moderately differentiated T3 N1 M0 adenocarcinoma of the rectum in 2015 with preoperative short course radiotherapy, anterior resection and then adjuvant chemotherapy with oxaliplatin and capecitabine. Following ileostomy reversal, he complained of intense, unremitting anorectal pain. After repeated scans, computed tomography (CT) showed findings suggestive of a longstanding anastomotic leak. Subsequent, magnetic resonance imaging (MRI) revealed osteomyelitis of the sacrum, with the development of sacral osteomyelitis in this context unusual. Our case highlights the importance of appropriate radiological imaging and that clinicians should consider osteomyelitis as a differential diagnosis in patients presenting with severe anorectal pain after treatment for rectal cancer.

## CASE REPORT

A 66-year-old man with a diagnosis of moderately differentiated T3 N1 M0 adenocarcinoma of the rectum in 2015 underwent short course radiotherapy prior to anterior resection that was followed by adjuvant chemotherapy with oxaliplatin and capecitabine, which were stopped prematurely due to grade 1 peripheral neuropathy. His temporary ileostomy was reversed 1 year later. Following reversal, he was troubled with on-going difficulties of bowel function including increased frequency, urgency with incontinence and tenesmus. Most troublesome of all was progressive rectal pain. He was referred to our Consequences of Colorectal Cancer Clinic, 4 years later.

**Figure 1:**
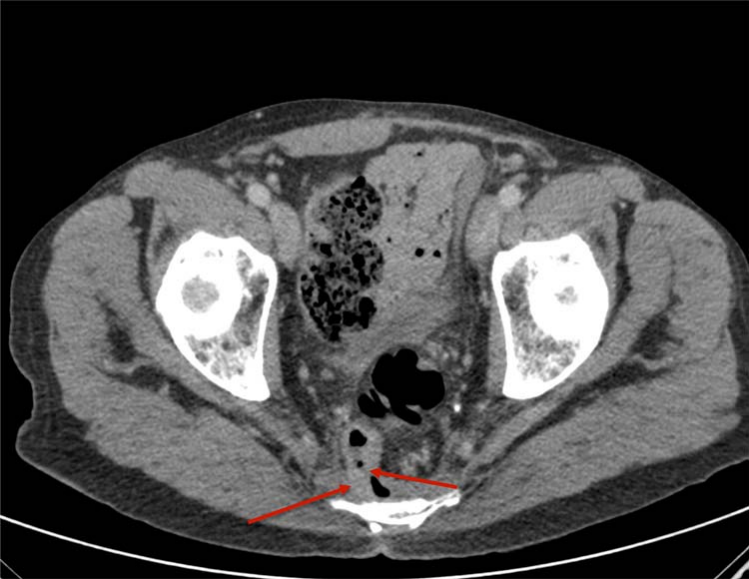
Axial section of contrast-enhanced CT (CECT) shows the communication of rectum to presacral collection at anastomotic site.

Flexible sigmoidoscopy showed no significant changes other than a previous anterior resection. His blood tests during this time showed normal leukocyte counts and a mildly elevated C-reactive protein that ranged from 16 to 29. Three CT scans of his abdomen and pelvis were arranged before referral to us. The initial CT in July 2017 showed presacral soft tissue thickening with air pockets that were in keeping with typical postsurgical changes. However, unusually, this presacral collection became more prominent in subsequent CT scans. By the time of his third CT in late 2018, a fistula between the rectum and the presacral collection at the anastomotic site could be identified (Fig. 1), suggesting a longstanding anastomotic leak. Multiple sinus tracts were also seen on this CT scan.

**Figure 2:**
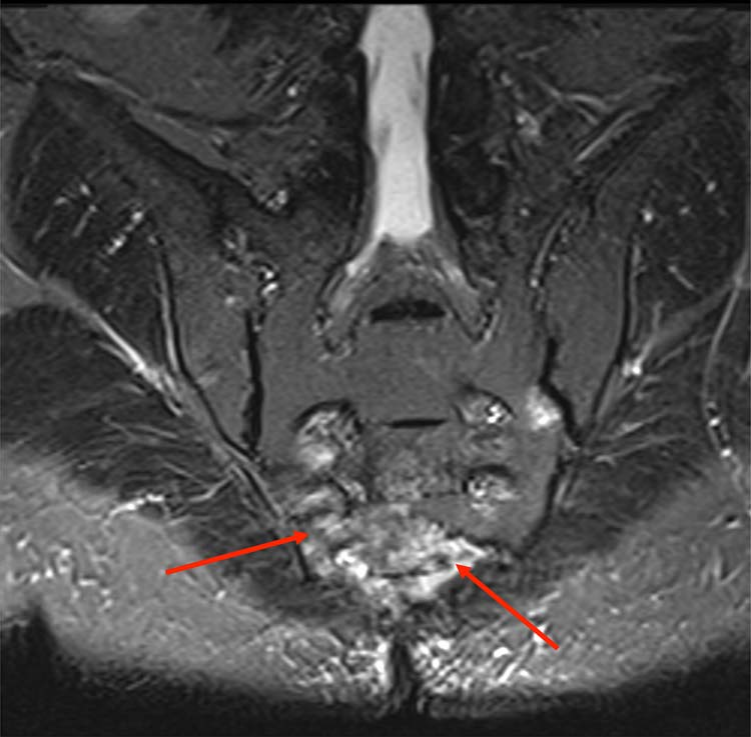
Short-TI Inversion Recovery coronal sequence showing hyperintense signal in lower sacral vertebrae representing changes due to chronic osteomyelitis.

Due to his on-going symptoms and for better delineation of the anatomic structures, MRI of the sacrum and coccyx with Short-TI Inversion Recovery sequences was requested. This showed hyperintense signalling in the lower sacral vertebrae representing changes consistent with chronic osteomyelitis (Fig. 2). The presacral collection was also again seen and consistent with a persistent small leak from his previous anterior resection. The patient was therefore discussed at the surgical MDT. The decision was made to treat him empirically with 6 weeks of intravenous ceftriaxone and oral metronidazole. There was no attempt made to obtain an aspirate from the collection. Once the antibiotic treatment was completed, the patient went on to have a Hartmann’s procedure performed. This led to very rapid reduction in and then abolition of his pain. On subsequent reviews, his stoma was functioning well and he had no rectal symptoms.

## DISCUSSION

Long-term significant defaecatory dysfunction following anterior resection is estimated to occur in between 37 and 90% of patients [1]. When anorectal pain is present, it may indicate local recurrence, anal fissuring [2] or rarely deep ulceration of ischaemic origin, either from the radiotherapy or injudicious endoscopic or local surgical intervention in irradiated mucosa. Radiotherapy-induced insufficiency fractures may also be a cause [3]. Occasionally, anorectal pain after pelvic radiation therapy is attributed to neuropathy; however, in our experience, this is very rare with modern radiotherapy techniques. Symptoms develop insidiously and are often dismissed when recurrence has been excluded.

Sacral osteomyelitis is an extremely rare complication of treatment for pelvic cancer. When it occurs, it is usually associated with fistulization from the rectum as in our patient or in one other case we have found [4] or from the urinary tract [5]. If left untreated, it can often be life-threatening due to the sequelae of sepsis, neurological involvement and fulminant faecal meningitis [4].

MRI imaging should be utilized to assess patients with ongoing anorectal pain following treatment for pelvic cancer. In those who have developed sacral osteomyelitis, MRI demonstrates changes in the water content of bone marrow while also providing excellent structural definition [6]. It also helps to plan for optimal surgical management and to assess the extent of devitalized tissue [7]. If MRI is contraindicated or unavailable, nuclear medicine studies and CT are useful alternatives. Nuclear medicine studies involve intravenous administration of a radionuclide, which emits radiation that is detected by a gamma camera. This allows assessment of abnormal bone metabolism, which in osteomyelitis manifests as areas of increased radionuclide uptake. In general, nuclear medicine studies have very high sensitivity in the detection of osteomyelitis and allow imaging of the whole skeleton to look for multiple sites of infection [8]. However, they are limited by poor specificity and anatomical localization. CT, on the other hand, can demonstrate clearly the anatomical relationship between areas of infection and important structures, such as adjacent bowel and major vessels. CT also has superior bony resolution to MRI and is better at demonstrating osseous changes such as cortical destruction, periosteal reactions and sequestrum formation. However, CT is unable to demonstrate bone marrow oedema, which means that a normal CT does not exclude early osteomyelitis [8].

Treatment of sacral osteomyelitis is often multi-modal and can involve a combination of targeted antibiotic therapy, radiological drainage and/or surgery [4]. It is therefore important to approach cases in a multi-disciplinary manner, with the involvement of an experienced radiologist and surgeon. The decision to surgically intervene must be individualized to the clinical condition of the patient, with more aggressive and extensive surgery recommended for those patients with significant sepsis and extensive bony or neurological involvement [4].

In conclusion, clinicians should have a low index of suspicion of an anastomotic leak and associated sacral osteomyelitis when treated rectal cancer patients present late, often after many years, with anorectal pain. Our case has highlighted the importance of this and how imaging can be utilized to diagnose the condition and plan for treatment.
